# Profiling Bacterial Diversity and Potential Pathogens in Wastewater Treatment Plants Using High-Throughput Sequencing Analysis

**DOI:** 10.3390/microorganisms7110506

**Published:** 2019-10-29

**Authors:** Cecilia Oluseyi Osunmakinde, Ramganesh Selvarajan, Bhekie B. Mamba, Titus A.M. Msagati

**Affiliations:** 1 Nanotechnology and Water Sustainability Research Unit, College of Science Engineering and Technology, University of South Africa, Florida Science Campus, Florida 1710, South Africa; coadebo@yahoo.com (C.O.O.); Mambabb@unisa.ac.za (B.B.M.); 2 College of Agriculture and Environmental Sciences, Department of Environmental Sciences, University of South Africa, Florida Science Campus, Florida 1710, South Africa; ramganesh.presidency@gmail.com

**Keywords:** high-throughput sequencing, bacteria, biodiversity, pathogens, wastewater treatment plant

## Abstract

Next-generation sequencing provides new insights into the diversity and structure of bacterial communities, as well as the fate of pathogens in wastewater treatment systems. In this study, the bacterial community structure and the presence of pathogenic bacteria in three wastewater treatment plants across Gauteng province in South Africa were studied. The physicochemical results indicated that the quality of wastewater varies considerably from one plant to the others. *Proteobacteria, Actinobacteria, Firmicutes*, and *Chloroflexi* were the dominant phyla across the three wastewater treatment plants, while *Alphaproteobacteria*, *Actinobacteria*, *Bacilli*, and *Clostridia* were the dominant classes. The dominant bacterial functions were highly associated with carbohydrate, energy, and amino acid metabolism. In addition, potential pathogenic bacterial members identified from the influent/effluent samples included *Roseomonas*, *Bacillus*, *Pseudomonas*, *Clostridium*, *Mycobacterium*, *Methylobacterium*, and *Aeromonas*. The results of linear discriminant analysis (LDA) effect size analysis also confirmed that these bacterial pathogens were significantly abundant in the wastewater treatment systems. Further, the results of this study highlighted that the presence of bacterial pathogens in treated effluent pose a potential contamination risk, transmitted through soil, agriculture, water, or sediments. There is thus a need for continuous monitoring of potential pathogens in wastewater treatment plants (WWTPs) in order to minimize public health risk.

## 1. Introduction

Waterborne infections emanate from the transmission of pathogenic microorganisms (bacteria, viruses, protozoa) by both direct and indirect ingestion of polluted water [1,2,3,4]. Most water-borne pathogens are spread through the fecal–oral route, and find their way to wastewater treatment plants (WWTPs) through excreted feces in raw sewage from infected people or in drinking water, or by eating food exposed to contaminated water, which could lead to serious health complications [4,5,6,7]. In recent years, bacterial pathogens have been recurrently identified in wastewater plants, based on the impression that WWTPs are a major reservoir for the growth of numerous pathogenic microbes [2,6,8]. Regardless of tremendous efforts by governments and water management facilities to improve and sustain the quality of water treatment systems, the occurrence of waterborne infections remains rampant worldwide [2,7]. Bacteria, which are abundant in WWTPs, play significant roles, such as breaking down organic matter, being a source of nutrients, and facilitating energy flow and biogeochemical cycling as well as disease transmission [9,10,11,12]. Traditionally, the main treatment processes in WWTPs include primary, biological (secondary), and tertiary treatment [13]. Primary treatment is a preliminary stage which makes use of bar screens to remove large solid objects from water. It can also include the use of sedimentation basins to separate solid from liquid waste by means of natural gravity. The floating materials are removed while the remaining liquids are laid open for the secondary stage of treatment through the use of biological methods (either aerobic or anaerobic) to break down the organic waste matter. The organic matter in the waste are removed through the use of a bed of stones (trickling filter beds) or the activated sludge process. Finally, effluents from the secondary treatment tank are then disinfected with chlorine, which is assumed to kill all pathogenic organisms and reduce odor, before being discharged into distribution channels and receiving water bodies. There has been a significant effort by water management authorities to adopt new techniques in treatment plants such as UV and membrane treatment technology, mechanical (ultrafiltration, reverse osmosis, nano-filtration), ultrasonic (ozonation), biological (active filters and enzymes), adsorption (activated carbon), and combinations of these processes to eliminate and partially remove microbes in wastewater [12,13,14,15,16,17,18,19]. Despite all these approaches, the existence of pathogenic microbes in raw and treated wastewater is still considered a potential hazard and health risk to the public [6,12,14]. At present, most WWTPs assess the quality of water based on biological indicators, such as the total coliforms and fecal coliforms [20], as well as physical and chemical indicators [13,21]. However, these assessment techniques do not directly classify the variety of the mixed bacterial populations present in WWTP systems.

Several molecular methods have been reported for the identification of bacterial species in environmental samples. There is, however, another challenge with regard to the species identification of microbes, in that a widespread selection of these environmental bacterial species cannot be cultured [22,23,24]. Over the years, molecular technologies such as polymerase chain reaction (PCR) [4,25,26,27,28], quantitative real-time PCR (qPCR) [4,28,29,30], microarray [4,31], and other techniques have been widely used to target microbial species in environmental samples [8,30]. All of these molecular techniques have both strengths and weaknesses, but these approaches are always delegated to specifically target the identification of a pre-selected group of microbes or virulence genes [4,25,31,32]. The main limitation of these technologies is that they can only specifically detect certain pathogens, and cannot provide comprehensive insight into the potential pathogens in the environment.

Current approaches in microbial detection (such as next-generation sequencing) have made it possible to extract DNA directly from environmental samples and sequences for identification, diversification, diagnostic, clinical, and monitoring purposes [33,34,35,36,37,38,39]. Recently, high-throughput next-generation sequencing methods applied to the 16S rRNA gene have produced complete descriptions of bacterial communities in various environments, due to the increased number of sequence reads that can be obtained and analyzed. A number of investigations have been performed into the microbial diversity and pathogenic indication of environmental genomes in WWTPs by sequencing of the 16S rRNA gene amplicon using different high-throughput techniques [2,7,9,11,40,41]. In this study, we investigated the abundance and diversity of the bacterial communities in influent and effluent wastewater samples from three WWTPs in South Africa, using a targeted amplicon-based sequencing approach. The study evaluated the potential biomarkers and abundance of bacterial communities that were removed after treatment processes to identify pathogens (including those not conventionally checked during quality assessment).

## 2. Materials and Methods

### 2.1. Sample Collection and Field Measurements

Wastewater samples were obtained from three different treatment plants in Pretoria, Krugersdorp near Johannesburg city. Daspoort wastewater treatment works (S 25°44′063″ E 028°10′688″), is located on the southern bank of the Apies River, northwest of the central business district in Pretoria, and it flows into dams and reservoirs from which it is withdrawn back to WWTPs; these flows will be referred to as DI for (influent) and DE (effluent). This plant is designed to treat a capacity of 60 million liters (60 megalitre per day) of wastewater. Samples were also collected from the Percy Stewart (PS; PSI: influent; PSE: effluent) (S 26°51′18.4″ E 27°52′57.2″) wastewater treatment plants located in Krugersdorp. The effluent from the plant is discharged into the Bloubankspruit River which connects with the Crocodile River and the surrounding Hartbeespoort Dam. The final samples were from Flip Human (FH; FHI: influent; FHE: effluent) (S 26°01′25.8″ and E 28°17′10.0″); its effluents are discharged into the neighboring Mooi River which flows to the Vaal Dam. The FH and PS treatment plants have an average daily flow capacity of 32 megalitre per day. All the treatment plants receive their waste mainly from households, industries, and hospitals in Pretoria, Krugersdorp in Johannesburg. The treatment processes in the three plants are quite similar but have different capacities. These comprise a coarse screen, fine screen, and a grit removal chamber for the removal of larger solids. The screened wastewater is then channeled into the primary sedimentation tank and a separate sludge digestion in rectangular tanks for the decomposition of waste, clarification, and then chlorination. All the studied plants make use of bio-filtration and biological nutrient removal (BNR) for the activated sludge system. The plants make use of bioreactors containing living material to capture and biologically degrade the organic matter. On the sludge side, a dissolved air flotation (DAF) thickening process is done to remove the suspended solids; dewatering of the sludge is accomplished using solar drying beds.

### 2.2. Physicochemical Analysis of Water Samples

From each sampling site (DE, DI, PSE, PSI, FHE, FHI), 2 L of raw (influent) and treated (effluent) wastewater samples from three different wastewater treatment plants were collected in triplicate in sterilized polypropylene bottles to prevent contamination and deterioration. The collected wastewater samples were preserved in ice-boxes and transported to the laboratory for further processing within 24 h. About 500 mL of each sample was stabilized with 5 mL nitric acid for heavy metal analysis, and the analysis was performed in an inductively coupled optical emission spectrometer (ICP-OES, Perkin Elmer, Waltham, MA, USA). The other half of the sample was filtered using 0.45 µm acrodisc^®^ syringe filters (PALL life sciences, NY, USA). Anions were determined using ion chromatography (chloride, fluoride, nitrite, bromide, nitrate, phosphate, and sulfate), while the dissolved organic carbon (DOC) in the samples was determined using the total organic carbon analyzer. For each sampling point, selected physicochemical parameters such as temperature, pH, dissolved oxygen, total dissolved solid (TDS), conductivity, and salinity were measured using the onsite multi-probe (YSI^TM^ 6 series, Sonde Marion, Germany) at the sampling site after collection.

### 2.3. Nucleic Acid Extraction and 16S-rRNA-Based Amplicon Sequencing

Prior to extraction, wastewater samples for molecular analysis were homogenized and filtered through a Whatman filter paper #114 (Sigma-Aldrich, USA) to remove big and coarse particles. The filtrate samples were then passed through a 0.20 µm Supor^®^ membrane filter (PALL Life Sciences, NY, USA) using a peristaltic pump to concentrate the microbial cells. Total genomic DNA was extracted from the concentrated samples using the Soil/Fecal DNA Extraction Kit™ (Zymo Research Corporation, USA) according to the manufacturer’s protocol. Total DNA was eluted and then quantified using a Qubit 3.0 Fluorometer (Life Technologies, Thermo Fisher Scientific, Waltham, MA, USA).

The extracted DNA was amplified with a set of primers targeting the hypervariable V1–V3 regions. The primers used were 27F (5′-AGAGTTTGA TCMTGGC-3′) and 518R (5′-GTATTACCGCGGCTGCTGG-3′), as described by Sibanda et al. [38]. The PCR amplification reactions contained 25 μL of one Taq 2X Master Mix, 22 μL of Nuclease-free water, and 1.5 μL of both forward and reverse primers at a concentration of 0.2 μmol/L with a 2 μL of extracted DNA (50–100 ng/μL) to make up a volume of 50 μL. The PCR conditions were set at 95 °C for 10 min for the initial denaturation, followed by 32 cycles of denaturation at 95 °C for 30 s, annealing at 55 °C for 30 s and extension at 72 °C for 1 min, and a final extension at 72 °C for 10 min, followed by cooling to 4 °C. All purified PCR products were then sequenced by paired-end sequencing chemistry, along with their multiplex sample identifiers on the Illumina MiSeq Platform (Inqaba Biotechnology, Pretoria, South Africa).

### 2.4. Data Processing and Bioinformatics Analysis

The quality control and filtering of the datasets were performed using MOTHUR pipeline version 1.40.0 [42]. The sequences were quality trimmed and paired-end joined using fastq-join to convert the overlapping paired-end reads into longer fragment sequences [43]. This was done to remove sequences with average quality scores of 8 nt, ambiguous bases, or >2 mismatches to primer sequences. The UCHIME algorithm was used to eliminate chimeric sequences using default parameters [44]. The non-chimeric (490 nt) sequence reads were aligned against the SILVA 16S rRNA gene reference database (version 128) with a confidence threshold of 80% [45,46]. The assignment of operational taxonomic units (OTUs) was performed at 97% for species identification using the furthest neighbor algorithm. Evaluation of the observable characteristic bacteria from each WWTP was achieved through the METAGENassist online software [47]. The highest OTUs at genus level were used to generate a phylogenetic heat map to visualize the dominant taxonomy for each WWTP sample and determine dissimilarity between samples’ bacterial communities. Statistical analysis—including one-way ANOVA and Turkey’s multiple range tests—were calculated to compare the mean values of the tested parameters for all the different sampling sites. Community diversity indices were calculated based on the OTUs obtained from the influent and effluent samples using nonparametric diversity indices. Chao1, the abundance-base coverage estimator (ACE), Shannon index (*H*), Simpson index (*D*), and Good’s coverage index were calculated at a distance of 0.03 using PAST (Paleontological statistics) [48]. To identify the potential biomarkers with significant differences between influent and effluent samples, the linear discriminant analysis (LDA) effect size (LEfSe) method was used [49]. Likewise, PICRUSt (phylogenetic investigation of communities by reconstruction of unobserved states) analysis was used to predict the functional abilities of identified bacterial communities in the wastewater samples using the KEGG (Kyoto Encyclopedia of Genes and Genomes) orthologues and pathway [49]. Breifly, the dominant OTU gene sequences, which represented the most abundant genes in the sample were mapped against the Greengenes database, using 97% confidence intervals to predict the presence of any functional gene in the sample [50]. The Ribosomal Database Project (RDP) classifier, using a method described previously by Wang et al. [51], was employed to identify possible pathogenic bacterial genera within the treatment plants.

## 3. Results

### 3.1. Physicochemical Profiles of the Wastewater Samples

**Figure 1:**
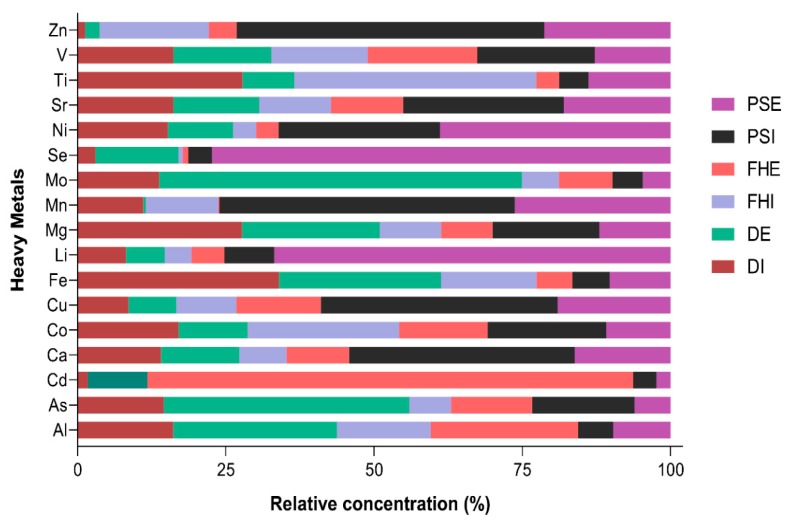
Heavy metal concentration levels (ppm) from the three WWTPs.

The selected physical and chemical characteristics of the wastewater samples (DI, DE, FHI, FHE, PSI, and PSE) are presented in Table 1. The temperature of the sampling points ranged from 15.6 to 21.2 °C, with an average temperature of 15.65 °C. Other physicochemical profiling parameters such as electrical conductivity (COND), salinity (SAL), DOC, dissolved oxygen (DO), and major anions noticeably varied among the wastewater samples. Greater variation of COND was observed in the water samples, with the values recorded ranging from influent (685.5–902 µScm^−1^) and effluent (506–1016 µScm^−1^). The total dissolved solid (TDS) measured in the influent samples varied from 341 to 513.3 mg/L, and from 253–508 mg/L in the effluent wastewater samples. The DOC, DO, salinity, and anions values varied among the influent and effluent samples. Statistically, the levels of pH, DO, DOC, TDS, SAL, COND, Cl^−^, F^−^, SO_4_^2−^, and PO_4_^3−^ were significantly higher (*p* < 0.05) in the studied influents of the WWTPs (Table 1). Furthermore, there were significant variations (*p* < 0.05) between the abovementioned parameters, except for F^−^ and DO, which were significantly lower (*p* > 0.05) in the effluent wastewater samples. Heavy metal concentrations in the wastewater samples were unequally distributed across the treatment plants (Figure 1). Samples PSI and PSE exhibited higher levels of Ca, Cu, Fe, Mn, Ni, and Zn; the DI and DE samples had higher concentrations of Fe, Mo, and Mg; and the FHI and FHE samples revealed high concentrations of Co only in both the influent and effluent samples.

### 3.2. Diversity Analysis for Bacterial Communities at the WWTPs

Bacterial diversity was analyzed based on the 16S rRNA amplicon sequence analysis, resulting in a total of 395,529 reads across the six samples, distributed as follows: 42,069 reads for sample DE, 33,958 for sample DI, 81,220 for sample FHI, 62,939 for sample FHE, 75,331 for sample PSI, and 100,012 reads for sample PSE. After OTU picking, we found a total of 1506 OTUs based on a 0.97 threshold across all samples. The microbial diversity indices were calculated, encompassing community richness (ACE, Chao1) and community diversity (Shannon and Simpson indices). The lowest OTUs were observed in sample FHE, while the highest OTUs were found in sample DI. Each sample had more than 99% the coverage, indicating that the depth of the sequence was sufficient. According to the OTU numbers, the sample from DI (519) had the richest diversity, followed closely by the PSE (351) and FHI (229) samples, whereas the samples from FHE (104), PSI (144), and DE (159) displayed considerably less richness. As is shown in Table 2, the values of the Ace, Chao, and Shannon indices demonstrated that the DI samples had the highest microbial diversity, while FHE had the lowest.

### 3.3. Taxonomic Composition of the WWTPs’ Microbial Communities

**Figure 2:**
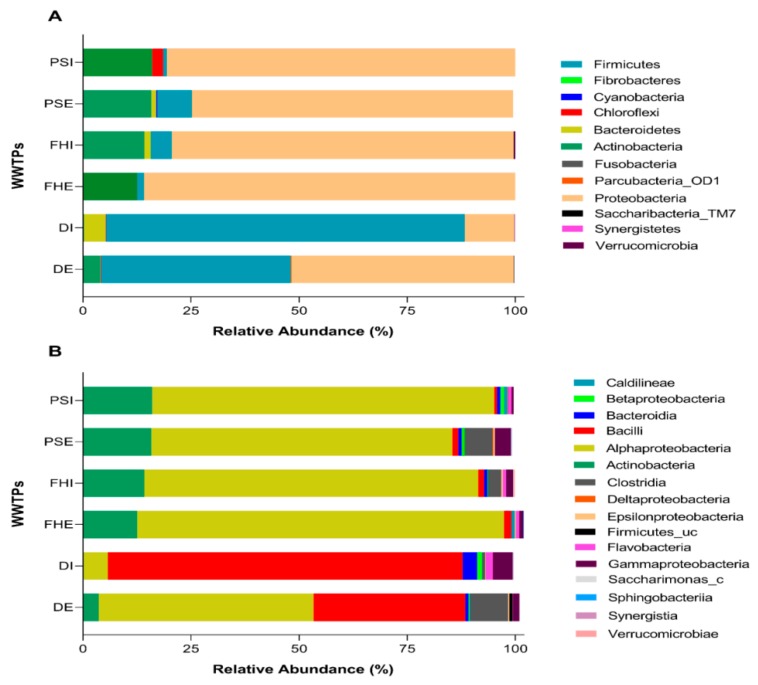
Bar charts showing (**a**) taxonomic distributions of dominant bacterial phyla and (**b**) taxonomic distributions of dominant bacterial classes for the six wastewater samples based on sequence data.

The relative abundances of bacterial groups in the different influent and effluent samples were analyzed at the phylum, class, and genus levels. Overall, a total of 17 bacterial phyla, 36 classes, 62 orders, 101 families, and 299 genera were identified from the six samples. The bacterial community structures and the relative abundance at the phylum and class levels, based on the dominant groups, are displayed in Figure 2a and Figure 2b respectively. As indicated in Figure 2a, at phylum level, the overall wastewater sequence reads were classified into three dominant phyla (relative abundance ≥1% in at least one sequence library), those being *Proteobacteria, Firmicutes*, and *Actinobacteria*. Samples FHE, FHI, PSE, PSI, and DE had the highest abundance of *Proteobacteria* (51.5%), while sample DI had the lowest abundance (11.4%). The influent sample DI had the highest abundance of *Firmicutes* (82.9%), DE had 43.9 %, while PSI contained the most Actinobacteria (16.5%). *Bacteroidetes* was only dominant in DI (6%), while *Chloroflexi* represented about 3.5% in PSI. At the class level, the dominant classes at the six wastewater samples were *Alphaproteobacteria, Actinobacteria, Bacilli*, and *Clostridia* (Figure 2b). *Alphaproteobacteria* were dominant in the majority of wastewater samples, including FHE, FHI, PSE, DE, and PSI, while in samples DI and DE, Bacilli was the dominant group. Other notable bacterial classes were *Gammaproteobacteria, Flavobacteria, Bacteroidia, Firmicutes, Deltaproteobacteria*, and *Betaproteobacteria*, which each contributed more than 1% to the total bacterial community in at least one sample.

**Figure 3:**
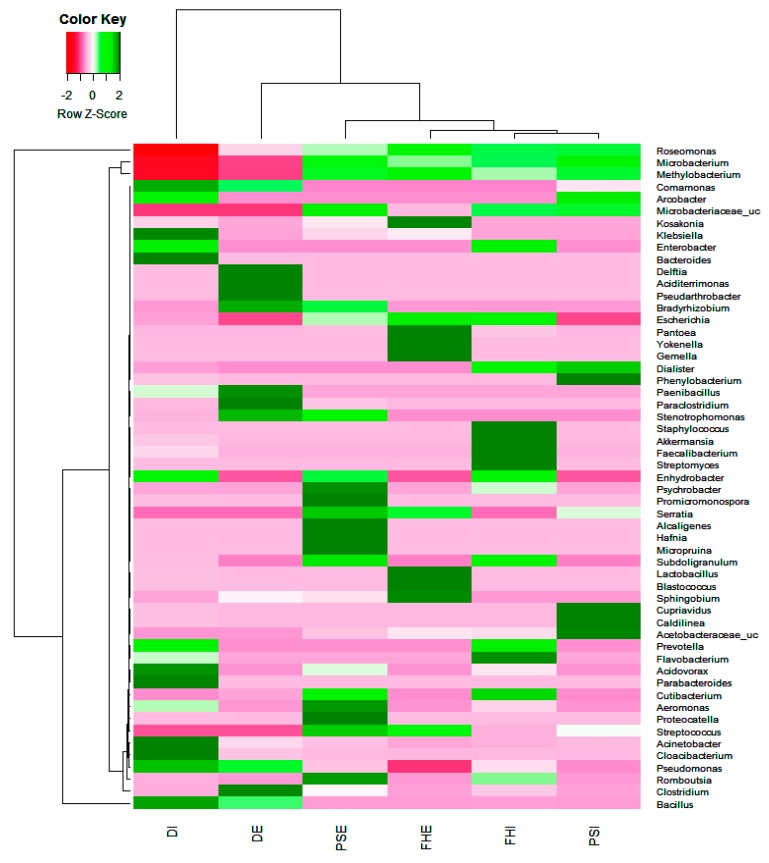
Heat map showing top 20 genera in the influent and effluent samples. The 20 dominant genera in each sample were selected and compared with their abundances (percentages) in other samples. The color intensity (log scale) in each panel shows the percentage of a genus in a sample, referring to a key.

At the genus level, 299 genera were acquired from all samples, with the top 60 being shared by all six samples; these accounted for about 60–88% of the classified sequences. The top 20 genera from the influent and effluent at the three WWTPs are shown in Figure 3. In total, more than 90% of the bacterial sequences from the samples were classified to the genus level. Among the 299 assigned genera, 14 were shared by all six samples; the influent and effluent samples were dominated by the genera *Bacillus, Sphingobium*, *Roseomonas*, *Propionibacterium*, *SMB53*, *Acinetobacter*, *Methylobacterium*, *Clostridium*, *Pseudomonas*, *Paenibacillus*, *Enhydrobacter*, *Prevotella*, *Streptococcus*, *Staphylococcus*, *Hafnia*, and *Microbacterium.* Rare genera were only observed in one or two samples, accounting for 1% of the total classified sequences in all six samples.

**Figure 4:**
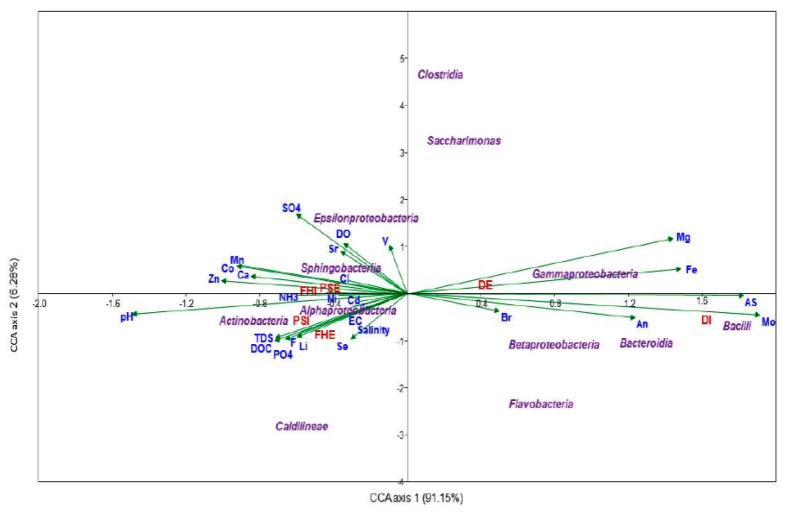
Canonical correspondence analysis (CCA) ordination plot shows the effect of physicochemical parameters on bacterial community structures (classes) in three different WWTPs.

A canonical correspondence analysis (CCA) bi-plot was used to illustrate the possible ecological relationships between microbial community and physicochemical variables within the WWTPs (Figure 4). CCA Axis 1 explained 91.15% of the variance, while Axis 2 explained 6.28% of the variance in the bacteria–environmental parameters relationship. In essence, the length of the arrow is proportional to the rate of change, so a longer arrow indicates a larger change in environmental variable. The CCA plot revealed strong relationships between the bacterial communities and measured physical and chemical water quality variables. Results of CCA revealed that the samples DI and DE were significantly different from other samples. In samples DI and DE, the distribution of the classes *Gammaproteobacteria*, *Betaproteobacteria*, *Bacteroidia*, and *Bacilli* was influenced by a combination of heavy metals such as Mg, Fe, Br, As, and Mo. The samples from the other treatment plants (PSE, PSI, FHE and FHI) exhibited high amounts of PO_4_, NH_3_, SO_4_, DO, DOC and metals (Ca, Mg, Zn, Cl, Ni); this significantly influenced the bacterial classes *Epsilonproteobacteria*, *Alphaproteobacteria*, *Actinobacteria* and *Sphingobacteria.* Members of *Flavobacterium*, *Saccharimonas*, and *Clostridium* did not show considerable impact from any of the physicochemical parameters observed in the wastewater treatment plants.

### 3.4. Significant Difference and Functions of Bacterial Communities in Influent and Effluent Samples

**Figure 5:**
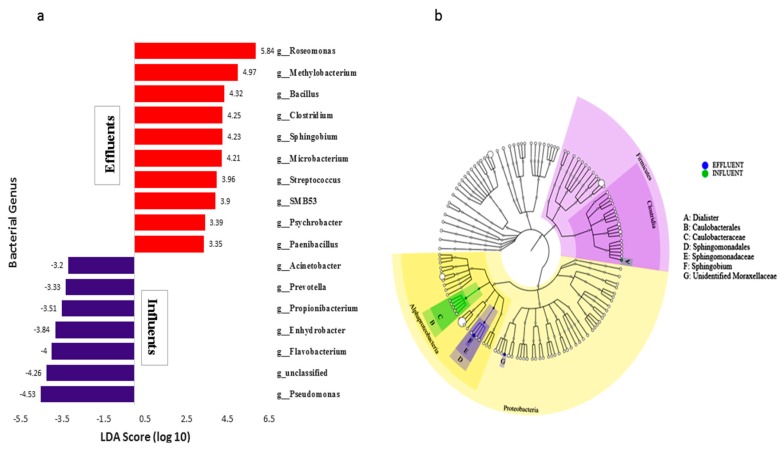
(**a**) Linear discriminant analysis (LDA) effect size (LEfSe) analysis showed differentially abundant genera as biomarkers determined using the Kruskal–Wallis test (*p* < 0.05), with LDA scores > 3.5. (**b**) Cladogram representation of the differentially abundant orders, families, and genera. The root of the cladogram denotes the domain Bacteria, and the size of each node represents its relative abundance.

To determine the classified bacterial taxa with significant abundance differences between the influent and effluent samples, the researchers performed biomarker analysis using linear discriminant analysis (LDA) effect size (LEfSe) analysis, as described by Segata and Huttenhower [49]. Differential features were identified at the OTU level (relative abundance > 1%). The nonparametric factorial Kruskal–Wallis rank sum test was used to detect taxonomies with significant differential abundances, while the LDA score was used to estimate the effect size of each differentially abundant trait, according to a method proposed by Zhang et al. [52]. LEfSe was able to compare the estimated phylotypes and identify the most differentially abundant taxa between the influent and effluent samples. As shown in Figure 5a significant differential abundances occurred between the collected influent and effluent samples. A cladogram for order, class, and genus level abundance is shown in Figure 5b. Comparison between effluent and influent samples identified 17 major genera: *Pseudomonas*, *Flavobacterium*, *Propionibacterium*, *Acinetobacter*, and *Enhydrobacter* were mainly enriched in the influent samples, while the genera *Roseomonas*, *Methylobacterium*, *Clostridium*, *Sphingobium*, *Microbacterium*, and *Bacillus* were significantly enriched within effluent samples, with LEfSe scores of 5.84, 4.97, and 4.32 respectively.

**Figure 6:**
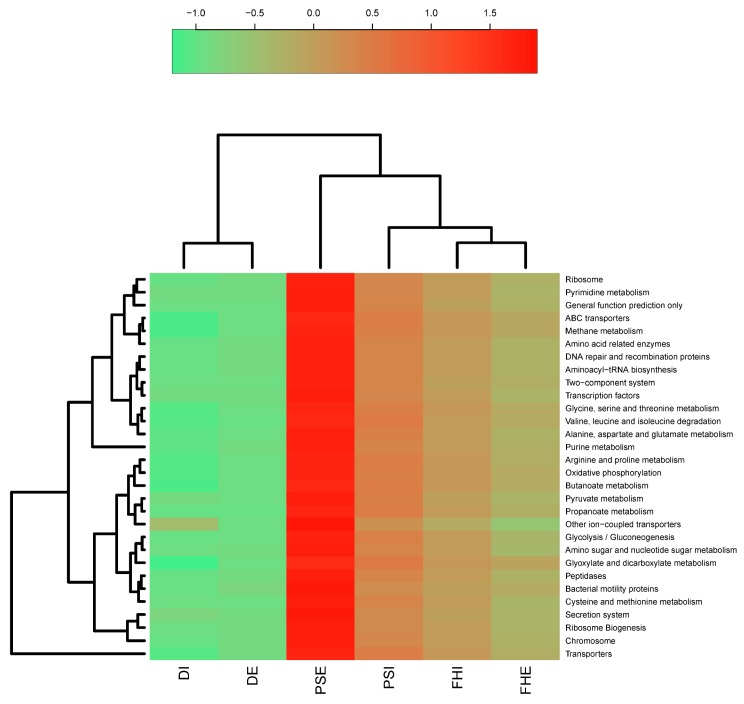
Heat map showing functional relative abundance in the WWTPs, predicted using PICRUSt (Phylogenetic investigation of communities by reconstruction of unobserved states) analysis.

Prediction of the metabolic functions from the 16S rRNA gene amplicon sequencing of the three different wastewater plants was achieved using PICRUSt analysis. The predictive functional profiles of the bacterial communities of the six wastewater samples are shown in Figure 6. The most abundant and prevailing classification was the metabolic pathway, which included carbohydrate, energy, and amino acid metabolism. The genes most associated with amino acid metabolic pathways were pyruvate metabolism, purine metabolism, histidine metabolism, alanine aspartate and glutamate metabolism, D glutamine and D glutamate metabolism, and arginine and proline acid metabolism. The results indicated that carbohydrate metabolism serves as a major energy source for other cellular processes. This also aligns with the fact that a major proportion of the WWTP relates mainly to the degradation of organic pollutants. Besides the metabolic pathways, genes responsible for genetic information processing were identified, which included ribosome biogenesis, transcription, and translation factors. Other important identified functional interactions included ABC (ATP-binding cassette) transporters, ion-coupled transporters, and DNA repair and recombination proteins (Figure 6).

### 3.5. Detection of Potential Pathogenic Bacterial Members

**Figure 7:**
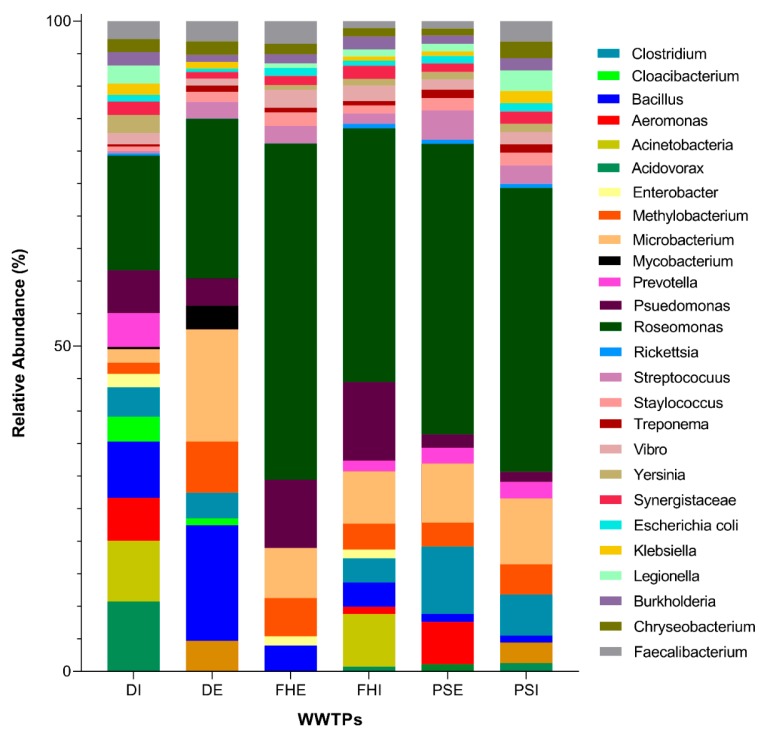
Potential pathogenic bacterial diversity and relative abundance at genus level at the WWTPs.

To investigate the presence of possible pathogens from the WWTP samples, the RDP classifier was used to identify pathogenic bacteria at the genus level. All the sequences identified as potentially pathogenic species were aligned using the local BLASTN tool; the read was identified as a potential pathogenic bacterium if it had an identity over 97% with its best BLAST hit. Most of the pathogenic bacterial 16S-rRNA-encoding DNA sequences were identified from the major phyla of Proteobacteria and Firmicutes. The results indicated that the pathogens in the WWTPs were relatively associated with 26 genera in the influent and effluent samples (Figure 7). At the genus level, the relative number of sequences apportioned to known pathogens was 10,863 (47.09%) for DI; 20,543 (81.23%) for DE; 24,018 (74%) for FHE; 21,925 (68.14%) for FHI; 2,817 (59.92%) for PSE; and 25,459 (69.29%) for PSI. The nine dominant genera *Roseomonas*, *Pseudomonas*, *Bacillus*, *Clostridium*, *Faecalibacterum, Methylobacterium*, *Micobacterim*, *Mycobacterium, Prevotella*, and *Aeromonas* were found to be dominant pathogens in all three WWTPs investigated. Among the genera identified, *Roseomonas* had the highest abundance in each wastewater sample, accounting for about 45% of all pathogenic sequences. Other genera, such as *Yersinia*, *Legionella*, *Burkholderia*, *Chryseobacterium*, *Treponema*, *Synergistaceae*, and *Escherichia* were also found to be over 1.35% in all samples, even after treatment.

## 4. Discussion

WWTPs play a significant role in the overall health of the ecosystem; however, diverse types of pollutants are being deposited into them on a day-to-day basis. The pH of a wastewater system is an important indicator of the quality of the water and the degree of pollution in the area. In this study, the pH of the influent wastewater samples varied between each plant, indicating that the wastewater samples were neutral to slightly alkaline, which seems to support the bacterial growth and activities required for WWTPs. Furthermore, the range of pH values of the treatment plants observed in this study was within the suggested limits determined by the South African Department of Water Affairs and Forestry in the National Water Act, 36 of 1998, the World Health Organization, and data reported elsewhere [53,54,55,56]. With respect to conductivity, high COND values were observed in all samples, which could be attributed to the presence of suspended impurities and dissolved ions in the water samples [57]. The concentration of heavy metals in the samples varied from one plant to the next, indicating the high presence of urbanization and industrial activities within the CBD environs [58,59,60]. Overall, the study indicated that heavy metals were unequally distributed across the treatment plants. The PSI and PSE samples revealed higher levels of Ca, Cu, Mn, Fe, Ni, and Zn. On the other hand, samples DI and DE had higher concentrations of Fe, Mo, and Mg, while the FHI and FHE samples revealed high concentrations of Co only, in both the influent and effluent samples. The detection of Zn, Ni, Fe, and other metals could have been the result of discharges from mine tailings, the extraction of metal ores, and erosion processes within the geographical location. Strengthening the monitoring and control of heavy metals at WWTPs can play a substantial role in improving environmental quality and reducing environmental risks associated with heavy metal pollution in water samples.

The current study investigated the bacterial diversity at three WWTPs by analyzing the OTUs, Chao1, and Shannon indices at cut-off levels of 97%. The results showed a slight difference in biodiversity among the influent and effluent samples. For example, the library derived from sample DI had the greatest number of OTUs (519), followed by samples PSE (351) and FHI (229), while sample FHE (104) had the smallest. Chao1 numbers were considerably higher than OTU numbers, which suggests that more OTUs may exist in these bacterial communities. However, no significant difference was observed in the Good’s coverage value of all samples (> 99%), which suggests that the observed sequences could function as a sound representation of the bacterial communities present in the six wastewater samples [61]. To assess the within-sample complexity of individual microbial populations, the Shannon–Weaver index (*H*) and evenness were calculated. The values of *H* ranged from 1.04 to 1.883 across the six wastewater samples, indicating that sample PSE (1.883) had the highest bacterial diversity and was highly species-rich. The OTUs and ACE observed in this study were much lower than those reported in previous studies [61,62], which found an average of 700 OTUs and 900 ACEs per sample identified.

*Proteobacteria* was the predominant phylum, constituting between 11 and 85% of all detected OTUs within the influent and effluent samples, which is consistent with the results from previous bacterial community studies in WWTPs [8,10,62,63]. *Firmicutes, Bacteriodetes, Actinobacteria, and Chloroflexi* were the subdominant groups, comprising 0.8–82.9%, 0.1–5%, 3.9–16.1%, and 0.1–2.5% of detections, respectively, within the six studied wastewater samples—which is consistent with previous studies [7,9,62]. In addition, a few low-abundance phyla accounted for more than 1% in at least one sample, such as *Verrucomicrobia* (0.2–1.3%), *Synergistetes* (0.1–1.8%), and *Saccharibacteria_TM7* (0.03–1.4%).

Within the Proteobacterial phylum, *Alphaproteobacteria* was the most abundant class, with FHE having the highest abundance of 85%, and DI having the lowest abundance (6%) across the wastewater samples. The studied WWTPs mainly deal with mixed wastes, domestic and industrial, and *Alphaproteobacteria* are known to be associated with bulking in industrial WWTPs [61]. A large population of *Alphaproteobacteria* has been detected in the WWTPs reported on in some others studies [61,63]. Thus, it can be established that *Alphaproteobacteria* is one of the prevailing bacterial species found in wastewater systems. Other prominent classes included *Bacilli*, with the highest abundance in DI (82%), *Actinobacteria* (2.8–10.0%), *Clostridia* (0.1–8.7%), and *Gammaproteobacteria* (0.5–5%). Flavobacteria had the lowest abundance in the wastewater samples, with values from 0.3–1.6%. The Proteobacterial members are the largest and most diverse in the Bacterial domain; they are mostly Gram-negative bacteria, which are of importance to human health, as well as having great metabolic diversity.

The dominant genera belonged to nine bacterial phyla, namely Actinobacteria, Spirochaetae, Firmicutes, Bacteroidetes, Chloroflexi, Verrucomicrobia, Acidobacteria, Acidovorax, and α, β, γ Proteobacteria. The most abundant genera were Methylobacterium, Roseomonas, and Pseudomonas, at 0.5–6.5% in individual samples, while Microbacterium, Propionibacterium, Actinomycetes, Bifidobacteriales, and Thermoleopilia were reported at an abundance of 1.5–3.8% in individual samples. Clostridium and Bacillus varied from 1.2–2.5%. The presence of these microbial species in WWTPs makes them potential targets for the bioremediation of some environmental pollutants [64,65]. Bioremediation involves the biological mechanisms of breaking down waste and pollutants into natural compounds and using them to restore the ecosystem to its original condition [65,66]. Studies have indicated that certain bacterial species can be used as bio-sorbents, which can help to degrade particular heavy metals [66,67,68,69], PCP [70], pesticides, poly-chlorinated biphenyl, and poly-aromatic hydrocarbons [67]. The additional use of microbe-based bio-sorbents for the elimination and recovery of toxic metals from industrial wastes can be an economical and active means of metal elimination. These results indicate that different WWTPs share a large proportion of their major bacterial populations, as these genera may play crucial roles in wastewater treatment regardless of geographic location.

The results from the LDA effect size (LEfSe) analysis identified several taxa that were most characteristic of the differences between the influent and effluent samples. Among those prevalent in the influent samples were members of the *Proteobacteria* (*Pseudomonas*, *Flavobacterium*, and *Enhydrobacter*), as well as some unclassified genera. The effluent samples were more dominated by the genera *Roseomonas*, *Methylobacterium*, and *Bacillus*, while traces of *Psychrobacter* and Paenibacillus were also observed. Many taxa contributed significantly to differences in the influent and effluent samples, including the fecal indicator members of the families of *Bacteroidetes*, *Enterobacteriaceae*, *Legionellaceae*, and *Arcobacter*.

The putative metabolic functions of the microbial communities at different depths were predicted through the use of the PICRUSt pipeline, which indicated mainly fermentation, fatty acid oxidation, glycolysis/gluconeogenesis, and methanogenesis. The estimated microbial metabolism did not differ noticeably between the effluent and influent samples. However, the most important predicted metabolic pathway was membrane transport. Different categories of membrane transport (ABC transporters) were predicted in both influent and effluent bacterial communities. The most common pathways predicted for carbohydrate metabolism were pyruvate metabolism, glycolysis/gluconeogenesis, and glyoxylate and dicarboxylate metabolism. Pyruvate plays an important role in breaking down glucose in prokaryotic cells: carbohydrates are converted through gluconeogenesis to fatty acids through a reaction with acetyl-CoA, which is then used to provide further energy to the cells [69]. Glucose is predicted to be converted into pyruvate by some bacterial species communities, which can then be used for cellular respiration. Another predicted form of energy metabolism in the bacterial communities was methane metabolism; this indicates that there may have been some Proteobacterial species with the biochemical potential of using methane as their source of energy [70]. *Verrucomicrobiae* have also been suggested to degrade polysaccharides [71]. This prediction was based on the consumption of propanoate and butanoate by bacterial communities, which denotes the oxidation of fatty acid and fermentation activities [71]. Several amino acid synthesis pathways were predicted, of which the most prominent were the ribosome, cysteine, methionine, alanine, valine, leucine, aspartate, and glutamate syntheses, isoleucine degradation, glycine, serine, threonine metabolism, and amino-acid-related enzyme pathways. On the other hand, the CCA confirmed that the presence of some heavy metals and anions was more relevant with respect to microbial diversity in wastewater. However, further investigations are needed to reveal the temporal dynamic relationships between microbial community composition and physicochemical factors.

Water is one of the most important natural resources on the planet, and is also critical for the continuity of life. Wastewater, however, contains certain toxic chemicals, organic and inorganic substances, and pathogenic or disease-causing microorganisms [7]. An investigation into the existence of indicator microorganisms in drinking water is fundamental for determining microbiological quality and public health safety. WWTPs play a fundamental role in reducing the microbial load of human waste pollutants before the final effluent is discharged to households or ecosystems, or as bio-solids to be used in agricultural activities [72].

The timely and prompt recognition of potential pathogens in the environment is one of the most crucial steps in averting the outbreak of infectious diseases, as drinking-water-related illness outbreaks still occur worldwide [72]. The 16S rRNA fingerprint has been used to identify those waterborne pathogens that have been indicated as priority pathogens by the WHO, which pose a significant health threat [53,55]. The pathogenic bacterial sequences identified in this study included some of the most common very pathogenic bacteria, which could be used for microbial source tracking, including *Roseomonas*, *Faecalibacterium*, *Bacillus*, *Aeromonas*, *Clostridium perfringens*, *Pseudomonas* *aeruginosa*, and *Escherichia coli* in both the influent and effluent samples. The highest abundance of potential waterborne pathogens was observed in samples DE, DI, and FHI, accounting for about 75.5% of all the OTUs. The phylum *Proteobacteria* was the most dominant, accounting for 47.1–80% in all the pathogenic bacterial compositions. This aligns with previous studies that detected Proteobacterial pathogens at sewage plants due to fecal contamination [2,7]. These results also point to the existence of environmental bacteria, enteric bacteria, and bacterial species being transmitted through wastewater [63]. Among the bacterial pathogens identified, the genus *Roseomonas* was dominant in all the wastewater samples. *Roseomonas* species are now gradually being referred to as evolving opportunistic pathogens for their connection to human infectious diseases [73,74,75], and are mainly observed in patients with compromised immune systems [74,75]. The occurrence of this genus in higher percentages indicates that hospitals in the area probably accommodate patients with different health histories (HIV, psychiatric, and cancer), judging from the higher values obtained. *Pseudomonas aeruginosa* was also prevalent across the samples; this bacterium has the potential to grow on the surface of any plastic pipe within the plant, thereby leading to infections in people with low immunity, and it is also resistant to many antibiotic drugs [1]. Other pathogenic bacteria identified at the WWTPs were *Chryseobacterium*, *Treponema*, *Staphylococuus*, *Streptococcus*, *Acinetobacteria*, *Aeromonas*, and *Faecalibacterium* in both the influent and effluent samples—a finding which is consistent with previous studies into the presence of these genera and species at treatment plants [2,7]. From a public health perspective, the presence of Aeromonas genus in the effluent sample is cause for concern, as is it associated with human disease outbreaks [76,77]. These pathogens are known to adhere to the surfaces of pipes and could cause antibiotic resistance [78]. *Escherichia coli* (*E. coli*) can cause severe foodborne diseases which cause fever, vomiting, nausea, abdominal pain, and diarrhea [1,79]. Other evident pathogens at WWTPs that can cause diseases in the intestines, include *Clostridium*, *Vibrio*, *Yersinia*, and *Faecalibacterium* [2,7], an indication of fecal contamination. Additional pathogenic bacteria which were noticed in the effluent samples and are cause for alarm—especially in communities which take their water directly from the river—were *Legionella*, *Bacillus*, *Klebsiella oxytoca*, *Burkholdria*, and *Mycobacteria*. These species cause fever, cough, chest pain, headaches, shortness of breath, and diarrhea. Disinfection at treatment plants with the use of chlorine is an extensively used technology that has proven to be efficient in inactivating a great variety of pathogenic microorganisms. It was observed that even after the disinfection stage at the WWTPs, pathogens were still present in the final effluents that are being released to drinking water distribution channels and receiving water bodies in significant amounts. This might be as a result of the ability of the inactivated pathogens to re-grow again in the disinfected sample. It could also be as a result of low doses of chlorine, or, most likely, the water effluent was not disinfected before being released into the environment and other distribution channels. Therefore, wastewater treatment controls the pathogens, but does not guarantee the complete elimination of pathogenic bacteria.

## 5. Conclusions

In conclusion, the present study reported the bacterial community structure from three WWTPs using a high throughput next generation sequencing approach. *Proteobacteria, Actinobacteria*, and *Firmicutes* were the dominant phyla in all treatment plants, while *Roseomonas* and *Enterobacter* was the dominant pathogenic bacterial genera. The results of this study highlight that high throughput sequencing provides comprehensive and accurate insight into environmental bacterial pathogens that other conventional techniques fail to indicate during water quality assessment. Indeed, NGS-based detection approaches have the potential to be interpreted into actionable data for water quality managers. A limitation with the use of the partial 16S rRNA approach is that there is a need for amplification step—this step could introduce some bias. This might affect the accurate picture of the bacterial communities actually present in the sample. In this study, the 16S rRNA approach was unable to identify new and divergent strains within the samples. There is still more work to be done in the development and application of metagenomic data analysis. Based on the observed findings, it is evident that pathogens can be dispersed via WWTPs and need to be removed, as they form the basis for environmental pathogen contamination and disease transmission. This poses a major threat to public health and water confidence levels, especially during water recycling and reuse in water-scarce situations in South Africa.

## Figures and Tables

**Table 1:** Physicochemical properties of influent and effluent water samples observed from three wastewater treatment plants (WWTPs). (Mean ± SD, *n* = 3).

Parameters	Influent				Effluent			
	DI	FHI	PSI	*p*-Value	DE	FHE	PSE	*p*-Value
**Temp °C**	21.2	15.9	18.4	<0.0001	19.9	15.6	18.5	0.0002
**pH**	6.64 ± 0.01	7.8 ± 0.01	7.2 ± 0.01	0.0028	7.05 ± 0.03	8.12 ± 0.06	7.2 ± 0.01	0.144
**DO mg/L**	0.44 ± 0.6	0.67 ± 0.1	0.48 ± 0.4	<0.0001	3.45 ± 0.4	3.04 ± 0.5	1.89 ± 0.8	0.0001
**EC µScm^−1^**	840.5 ± 2.1	902 ± 5.6	685.5 ± 6.4	<0.0001	506 ± 0.01	774 ± 2.8	1016 ± 7	0.0085
**Salinity µg/L**	0.42 ± 0.01	0.45 ± 0.01	0.34 ± 0.01	<0.0001	0.25 ± 0.01	0.38 ± 0.01	0.51 ± 0.01	<0.0001
**NH_3_–N**	0.04 ± 0.01	0.17 ± 0.04	ND	0.0001	0.06 ± 0.01	0.13 ± 0.04	0.04 ± 0.01	<0.0001
**TDS mg/L**	420.5 ± 0.7	451.5 ± 4	513.3 ± 18	0.0003	253.0 ± 0.1	387.5 ± 2	508.0 ± 4.2	<0.0001
**DOC mg/L**	20.39 ± 0.62	55.13 ± 0.8	154.93 ± 2.5	0.0001	5.59 ± 0.7	11.73 ± 0.7	18.16 ± 0.9	0.033
**Cl^−^ mg/L**	28.64 ± 4.75	21.54 ± 0.6	60.57 ± 0.01	<0.0001	41.31 ± 0.34	19.34 ± 1.0	44.08 ± 2.0	<0.0001
**F^−^ mg/L**	0.23 ± 0.09	0.13 ± 0.01	16.97 ± 0.5	0.0003	ND	0.20 ± 0.01	0.20 ± 0.01	<0.0001
**Br^−^ mg/L**	0.54 ± 0.1	0.91 ± 0.1	ND	0.0003	ND	ND	ND	-
**SO_4_ mg/L**	6.68 ± 1.0	26.98 ± 0.1	3.51 ± 0.1	<0.0001	40.49 ± 0.5	22.91 ± 1.3	58.11 ± 1.4	0.0002
**PO_4_ mg/L**	5.07 ± 0.6	12.72 ± 0.1	23.29 ± 0.1	<0.0001	ND	0.71 ± 0.03	4.33 ± 0.1	0.0085

ND: not detected, (*p* < 0.05) defined as statistically significant.

**Table 2:** Summary for the bacterial richness and diversity of microbial communities for the WWTPs.

Parameters	DI	DE	FHI	FHE	PSI	PSE
**Quality reads**	25,287	23,066	32,176	32,469	36,742	46,924
**Average read length**	518	491	471	469	471	473
**OTUs**	519	159	229	104	144	351
**Good’s coverage %**	99.90	99.40	99.90	99.90	100	99.90
**ACE**	659.6	179.8	257.5	116.5	150.1	369.2
**Chao1**	620.7	170	240.2	110.1	146.2	357.3
**Shannon_H**	1.304	1.524	1.523	1.04	1.198	1.883
**Simpson_1-D**	0.663	0.347	0.484	0.568	0.51	0.383
